# Respiratory parameters on diagnostic sleep studies predict survival in patients with amyotrophic lateral sclerosis

**DOI:** 10.1007/s00415-021-10563-0

**Published:** 2021-04-20

**Authors:** Markus Engel, Christian Glatz, Cornelia Helmle, Peter Young, Bianca Dräger, Matthias Boentert

**Affiliations:** 1 Department of Neurology with Institute of Translational Neurology, University Hospital Münster, Münster, Germany; 2 Department of Neurology, Medical Park Reithofpark, Bad Feilnbach, Germany; 3 Department of Medicine, UKM Marienhospital Steinfurt, Steinfurt, Germany

**Keywords:** Amyotrophic lateral sclerosis, Sleep-disordered breathing, Non-invasive ventilation, Survival, Nocturnal hypercapnia, Base ecxess

## Abstract

**Objective:**

In amyotrophic lateral sclerosis (ALS), respiratory muscle involvement and sleep-disordered breathing relate to worse prognosis. The present study investigated whether respiratory outcomes on first-ever sleep studies predict survival in patients with ALS, specifically taking into account subsequent initiation of non-invasive ventilation (NIV).

**Methods:**

From patients with ALS, baseline sleep study records, transcutaneous capnometry, early morning blood gas analysis, survival data and clinical disease characteristics were retrospectively analyzed. Patients were stratified according to whether enduring NIV was consecutively established (“NIV(+)”) or not (“NIV(–)”).

**Results:**

Among the study cohort (*n* = 158, 72 female, 51 with bulbar onset ALS, 105 deceased) sleep-disordered breathing was present at baseline evaluation in 97 patients. Early morning base excess (EMBE) > 2 mmol/l predicted nocturnal hypercapnia. Ninety-five patients were NIV(+) and 63 were NIV(–). Survival from baseline sleep studies was significantly reduced in NIV(–) but not in NIV(+) patients with nocturnal CO_2_ tension ≥ 50 mmHg, apnea hypopnea index ≥ 5/h, and EMBE > 2 mmol/l. Hazard ratio for EMBE > 2 mmol/l was increased in NIV(–) patients only, and EMBE independently predicted survival in both NIV(–) and NIV(+) patients. Furthermore, EMBE on baseline sleep studies was the only predictor for survival from symptom onset, and hazard ratio for shorter survival was markedly higher in the NIV(–) than the NIV(+) group (2.85, *p* = 0.005, vs. 1.71, *p* = 0.042).

Interpretation: In patients with ALS, EMBE > 2 mmol/l predicts nocturnal hypercapnia and shorter survival. Negative effects of sleep-disordered breathing on survival are statistically abolished by sustained NIV.

**Supplementary Information:**

The online version contains supplementary material available at 10.1007/s00415-021-10563-0.

## Introduction

Amyotrophic lateral sclerosis (ALS) is a neurodegenerative disease mainly involving the motor pathways [1]. Median survival time is 2.5–3.5 years after symptom onset and 1.5–2.5 years following diagnosis [2, 3]. Prognosis is mainly determined by phrenic nerve involvement that puts patients at risk of chronic respiratory failure and pulmonary infections. These comprise the most frequent causes of premature death in affected patients [4, 5]. Mechanical ventilation has been shown to significantly improve health-related quality of life and survival [6, 7]. Moreover, early initiation of non-invasive ventilation (NIV) has proven benefits in patients with both non-bulbar and bulbar onset of disease [8]. Since respiratory failure first manifests as nocturnal hypoventilation [9], sleep studies and sensitive detection of sleep-related hypercapnia is essential for early implementation of NIV [10, 11]. In ALS, sleep-disordered breathing (SDB) may also encompass obstructive sleep apnea (OSA) and, rarely, central sleep apnea [10]. Measures of respiratory muscle dysfunction and SDB have been introduced as predictors of disease progression and overall prognosis [12–17]. However, the predictive value of nocturnal capnometry has not yet been evaluated, and only one study focused on the apnea hypopnea index (AHI) [14]. A recent study showed that daytime arterial base excess relates to respiratory muscle weakness and risk of death or tracheostomy [18]. The present study investigated whether nocturnal transcutaneous carbon dioxide tension, AHI, and early morning base excess (EMBE) on baseline sleep studies predict survival, taking into account whether NIV was subsequently initiated, and whether it was effective in terms of treatment adherence and sustained correction of SDB. The latter aspect has been proven meaningful in ventilated patients with neuromuscular disorders (including ALS) as persisting obstructive events, desaturations or nocturnal hypercapnia all impact survival [19–21].

## Patients and methods

### Patients and study design

We retrospectively analyzed clinical records and sleep studies derived from patients with ALS admitted for first-ever evaluation of sleep-related breathing between January 2010 and March 2018. All patients met the revised El Escorial criteria for possible, probable or definite ALS [22]. Patients underwent diagnostic sleep studies for the following reasons: FVC < 70% of the predicted value or symptoms possibly indicating sleep-disordered breathing, such as non-restorative sleep, sleep disturbances, morning headache, or daytime sleepiness. Subjects with any kind of mask-based therapy or invasive ventilation at initial presentation were excluded. The initial cohort comprised 285 patients. In 25 subjects sleep records were incomplete, 16 patients subsequently underwent tracheostomy, and for 86 patients follow-up data on later start of ventilatory support, health status or date of death could not be retrieved. Finally, 158 individuals entered survival analysis which focused on survival time after self-reported symptom onset (referred to as T0) and following baseline evaluation of sleep-related breathing (referred to as T1). As an endpoint for both timespans, T2 was defined as the date of death for deceased patients or the date of the last clinical status report for patients who were still alive when the database was closed. Reasons for exclusion of patients who had been tracheotomized at any time following T1 will be outlined in the discussion section. The study was approved by the local ethics authority (Ethikkommission der Westfälischen Wilhelms-Universität Münster und der Ärztekammer Westfalen-Lippe, 2016-178-f-S).

### Sleep studies

At baseline, either cardiorespiratory polygraphy (Weinmann, Hamburg, Germany; *n* = 83) or full polysomnography (Nihon Kohden, Rosbach, or Somnomedics, Randersacker, Germany; *n* = 75) was performed. Scoring of sleep and respiratory events followed standard recommendations [23, 24]. Respiratory sleep outcomes comprised oxygen desaturation index (ODI), AHI, peripheral oxygen saturation (SpO_2_), and duration of SpO_2_ falling below 90% ($t_{{{\text{SpO}}_{2} }}$_<90_). Sleep apnea was defined as AHI ≥ 5/h. Baseline, maximum and mean carbon dioxide tension (*p*_tc_CO_2_) were extracted from transcutaneous capnometry recordings that were available in all patients (Sentec, Therwil, Switzerland). Nocturnal hypoventilation was defined as peak *p*_tc_CO_2_ ≥ 50 mmHg (6.7 kPa) or an increase of 10 mmHg or more from the awake baseline value [25]. Cumulative duration of the *p*_tc_CO_2_ increase ≥ 50 mmHg ($t_{{{\text{CO}}_{2} }}$_≥ 50_) was specifically recognized as it has been proposed for defining nocturnal hypoventilation more recently [26]. We defined SDB as the presence of sleep apnea or nocturnal hypoventilation, or both. In 146 patients capillary blood gas analysis was available. Blood samples were taken from the arterialized earlobe within 1 h after awakening.

### Ventilator settings

Treatment settings comprised air humidification, nasal or oronasal interface, and pressure-controlled bi-level ventilation using a spontaneous-timed mode with average volume assured pressure support (AVAPS^®^, Philips Respironics). Following internal standards, expiratory positive airway pressure (EPAP) was set at 4 cmH_2_O if the AHI was < 5/h, at 6 cm H_2_O if the AHI was 5–15/h and 8 cmH_2_O in case of AHI > 15/h. Tidal volume, respiratory rate, and airway pressures were individually titrated to achieve normocapnia (*p*_tc_CO_2_ < 50 mmHg), normoxia ($t_{{{\text{SpO}}_{2} }}$_<90_ < 5 min) and normalization of the AHI (< 5/h). In a subset of patients, persistent obstructive events required implementation of a variable EPAP. Follow-up sleep studies including transcutaneous capnometry were scheduled every 3–6 months, and ventilator settings were adjusted, if necessary, to reach the above target values.

### Clinical measures and formation of subgroups

From clinical records, we collected demographic data, body mass index, and date and type of symptom onset (bulbar or non-bulbar). Baseline spirometry was available for 103 patients. Clinical status was documented using the revised ALS Functional Rating Scale (ALS-FRS-R) in 151 patients [27]. Presuming that the ALS-FRS-R score had been 48 prior to disease onset, we calculated the individual progression rate defined as the average monthly decline of the ALS-FRS-R score before admission to the sleep laboratory (ΔFS = 48 − ALS-FRS-R/duration from disease onset in months) [28, 29]. Regarding functional deterioration, patients were stratified as “slow” (ΔFS < 0.47/month), “moderate” (ΔFS 0.47–1.1/month) and “fast” progressors (ΔFS > 1.1/month) [29]. Patients were also categorized according to bulbar function at the time of baseline sleep studies using the bulbar subscore of the ALS-FRS-R. A subscore of > 6 was classified as ‘no, mild or moderate’ bulbar dysfunction and ≤ 6 was defined as ‘severe’ bulbar dysfunction [12]. Cognitive and behavioral impairment or presence fronto-temporal dementia (ALS-FTD) was documented according to current diagnostic criteria [30].

Data on NIV initiation, adherence to treatment, tracheostomy, survival status or date of death were collected from clinical records or obtained by contacting the patients’ caregivers and the deceased patients’ dependents. In patients using NIV, information on treatment adherence was specifically available from device memory data. Patients were subdivided in a NIV(+) group that comprised all patients with regular use of NIV until T2, and a NIV(–) group encompassing subjects in whom endurin*g* NIV was never established. The latter group included patients who either declined NIV, who were started on continuous positive airway pressure (CPAP) therapy only, or who transiently attempted but then abandoned NIV. Mean survival times and hazard ratios were calculated for different strata which were formed according to cut-off values for AHI (≥ 5/h), maximum *p*_tc_CO_2_ (≥ 50 mmHg), $t_{{{\text{CO}}_{{2}} }}$_≥50_ (≥ 30 min), EMBE (> 2 mmol/l)[22], and FVC (< 70% predicted) [26].

### Statistical analysis

Statistical data analysis was performed using IBM SPSS® 26.0 (IBM, Armonk, NY, USA). Results are presented as mean and standard deviation or standard error, respectively. For comparison of categorical variables the Chi-square test was applied. Comparison between means was performed using the two-tailed t test and ANOVA in case of normal distribution or the Mann–Whitney *U* and Kruskal–Wallis test for non-parametric data. Correlations between continuous variables were analyzed using Pearson’s or Spearman’s correlation coefficient as appropriate [31]. Cumulative 5-year survival was visualized using Kaplan–Meier plots and analyzed using the log rank test. Hazard ratios were calculated using Cox regression analysis with upright FVC, AHI, *p*_tc_CO_2_, $t_{{{\text{CO}}_{{2}} }}$ > 50, EMBE and ALS-FTD included. Effects of different variables on survival were analyzed using a linear regression model (including respiratory measures and the presence of ALS-FTD). *p* values < 0.05 were considered statistically significant. For multiple testing, Bonferroni’s correction was applied.

## Results

### Clinical characteristics of patients

Demographic data and disease characteristics at baseline are depicted in Table 1. One individual was diagnosed with familial ALS (SOD1 gene mutation). Ten subjects fulfilled diagnostic criteria of ALS-FTD [30]. Comorbidities included arterial hypertension (*n* = 47), chronic obstructive pulmonary disease (*n* = 3), and congestive heart failure (*n* = 1). Medication with riluzole was specified by the majority of patients at T1, with no significant difference between the NIV(+) and NIV(–) groups (Table 1). The number of patients with percutaneous gastrostomy increased from 10 at T1 to 54 at T2.

**Table 1:** Demographic data and disease characteristics in the entire study cohort and in patients stratified according to the presence of sleep-disordered breathing (SDB, columns 3 + 4) and enduring non-invasive ventilation (NIV, columns 6 + 7)

	All patients (*n* = 158)	SDB (*n* = 97)	No SDB (*n* = 61)	*p*	NIV(+) (*n* = 95)	NIV(−) (*n* = 63)	*p*
Male [n/%]	86/54.4	63/64.9	23/37.7	0.001^#^	54/56.8	32/50.8	N.S.^#^
Age at T0, [years]	61.4 (10.9)	63.2 (10.2)	58.6 (11.4)	0.009*	62.3 (10.1)	59.9 (12.0)	N.S.*
Age at T1 [years]	63.9 (10.4)	65.9 (9.3)	60.9 (11.3)	0.003*	64.6 (9.9)	63.9 (11.1)	N.S.*
Disease duration (T0–T1) [months]	30.2 (33.4)	31.9 (37.6)	27.6 (28.7)	N.S.^§^	26.9 (21.7)	35.2 (47.4)	N.S.^§^
BMI [kg/m^2^]	24.8 (4.1)	25.3 (3.9)	24.2 (4.4)	N.S.*	25.3 (4.3)	24.1 (3.8)	N.S.*
Deceased [n/%]	105/66.5	67/69.1	38/62.3	N.S.^#^	71/74.7	34/54.0	0.007^#^
Bulbar onset [n/%]	51/32.3	30/30.9	21/34.4	N.S.^#^	28/29.5	23/36.5	N.S. ^#^
Severe bulbar dysfunction at T1 [n/%]	41/25.9	25/25.8	16/26.2	N.S.^#^	24.0/25.2	17/27.0	N.S.^#^
ALSFRS-R total score at T1	35.6 (7.4)	35.0 (7.3)	36.5 (7.7)	N.S.^§^	35.3 (7.3)	36.0 (7.7)	N.S.^§^
ΔFS	0.8 (1.0)	0.9 (1.0)	0.8 (0.8)	N.S.^§^	0.9 (1.1)	0.7 (0.8)	N.S.^§^
Medication with riluzole at T1[n/%]	123/78.8	74/76.3	49/80.3	N.S.^#^	75/78.9	48/76.2	N.S.^#^
Survival time after T0 [months]	52.4 (40.8)	52.5 (43.9)	52.2 (35.8)	N.S.^§^	50.8 (31.1)	54.8 (52.4)	N.S.^§^
Survival time after T1 [months]	22.1 (18.9)	20.6 (17.7)	24.6 (20.5)	N.S.^§^	23.8 (20.4)	19.6 (16.1)	N.S.^§^

*T0* self-reported symptom onset, *T1* date of baseline sleep studies, *ΔFS* monthly loss on the ALS-FRS-R between T0 and T1, *N.S.* not significant

Numbers are depicted as mean and standard deviation (in brackets), or absolute number/percentage (in relation to the subgroup specified at the top of the columns)

*p* values ≤ 0.05 were considered significant

^#^Chi-square test

*t test

^§^Mann–Whitney *U* test

### Baseline respiratory parameters

Diagnostic sleep studies revealed SDB in 97/158 patients (61.4%) comprising isolated nocturnal hypoventilation in 17/158 subjects (10.8%), isolated sleep apnea with predominant OSA in 42/158 individuals (26.6%), and a combination of both in 38/158 patients (24.1%). Predominant OSA was defined by more than 50% of all apneas being obstructive in nature. Central sleep apnea was rare with only 8 patients showing a central apnea index > 5/h. In patients with blood gas analysis available, EMBE was > 2 mmol/l in 80/146 patients (54.8%). Among these, 26 patients (32.5%) had no SDB, and in 39/93 (41.9%) patients with SDB, EMBE was ≤ 2 mmol/l. In the entire cohort, EMBE was significantly associated with maximum *p*_tc_CO_2_ (*r* = 0.647, *p* < 0.001) and $t_{{{\text{CO}}_{{2}} }}$_≥ 50_ (*r* = 0.478, *p* < 0.001) but not with AHI (*r* = 0.091, *p* = 0.273). Linear regression revealed EMBE as an independent predictor of nocturnal hypoventilation with AHI, predicted FVC, mean SpO_2_, $t_{{{\text{SpO}}_{2} }}$_<90_ and disease duration at T1 forced into the model (*β* = 0.299, *p* = 0.007).

Male patients were affected more severely and more often by SDB (Table 2). Nocturnal hypoventilation was present in 36/86 (41.9%) of men and in 19/72 (26.4%) of women (*p* = 0.042). For OSA, prevalence numbers were 52/86 (60.5%) and 28/72 (38.9%), respectively (*p* = 0.007). Whereas pCO_2_ on morning blood gas analysis was higher in men than in women (albeit normal), EMBE showed no statistical difference between genders (Table 2). When patients were grouped according to severity of bulbar symptoms or disease progression as reflected by the ΔFS, FVC was significantly lower in patients with severe bulbar dysfunction, and EMBE was higher in fast progressors than in slow and moderate progressors (Table 2). Other respiratory sleep outcomes did not show significant differences between these subgroups (Table 2).

**Table 2:** Baseline respiratory parameters and survival in patients stratified according to gender, bulbar function, and progression rate

	All patients (*n* = 158)	Females (*n* = 72)	Males (*n* = 86)	*p*	Moderate, mild or no bulbar dysfunction (*n* = 117)	Severe bulbar dysfunction (*n* = 41)	*p*	Slow progressors (*n* = 69)	Moderate progressors (*n* = 49)	Fast progressors (*n* = 33)	*p*
AHI [1/h]	8.6 (11.6)	5 (5.9)	11.6 (14.1)	< 0.001^§^	9.8 (13)	5.2 (4.8)	N.S.^§^	9 (13.2)	9.3 (10.9)	7.1 (10)	n.s.^§^
ODI [1/h]	7.9 (9.4)	4.9 (6)	10.4 (10.9)	< 0.001^§^	8.7 (10.2)	5.7 (6.3)	N.S.^§^	8.3 (9.4)	8.8 (10.1)	6.2 (9)	n.s.^§^
Mean SpO_2_ [%]	93.3 (2.9)	93.9 (2.4)	92.8 (3.1)	0.018^#^	93.2 (2.9)	93.4 (2.8)	N.S.^#^	93.5 (2.9)	93.1 (3.1)	93.2 (2.7)	n.s.^#^
Minimum SpO_2_ [%]	83.5 (7)	84.5 (7.1)	82.7 (7)	N.S.^#^	83.4 (6.8)	84 (7.8)	N.S.^#^	83.9 (7)	82.2 (7.7)	84.9 (6.4)	n.s.^#^
$t_{{{\text{SpO}}_{2} }}$_<90_ [min]	49.3 (96.3)	27.5 (60.6)	67.1 (115)	0.010^§^	52.6 (97.6)	39.8 (92.9)	N.S.^§^	40.2 (98.5)	65.8 (108.6)	45.3 (77)	n.s.^§^
Maximum *p*_tc_CO_2_ [mmHg]	46.7 (6.6)	44.7 (5.6)	48.4 (6.9)	< 0.001^#^	46.7 (6.7)	47 (6.5)	N.S.^#^	46.5 (6)	47 (7.5)	47.3 (6.9)	n.s.^#^
Mean p_tc_CO_2_ [mmHg]	41.8 (5.6)	40.1 (4.3)	43.2 (6.1)	< 0.001^#^	41.8 (5.8)	41.8 (4.9)	N.S.^#^	41.8 (4.9)	41.7 (6.2)	42.4 (6.4)	n.s.^#^
$t_{{{\text{CO}}_{{2}} }}$_>50_ [min]	31 (100)	10 (51)	49 (126)	0.018^§^	34 (105)	24 (86)	N.S.^§^	24 (85)	43 (118)	34 (111)	n.s.^§^
*p*CO_2_ [mmHg]	39.6 (5.4)	38.3 (4.3)	40.7 (5.9)	0.007^#^	39.8 (5.6)	39.2 (4.8)	N.S.^#^	39 (4.8)	39.7 (5.5)	41 (6.6)	n.s.^#^
EMBE [mmol/l]	2.7 (2.6)	2.4 (2.6)	2.9 (2.7)	N.S.^#^	2.5 (2.7)	3.2 (2.4)	N.S.^#^	1.8 (2.2)	3.2 (2.5)	3.6 (3.1)	0.003^#^
FVC [% predicted]	68.8 (27.9)	66.1 (27.8)	70.8 (28)	N.S.^#^	72.1 (26.5)	50.8 (29.3)	0.006^#^	76.1 (26.6)	61.4 (31.2)	63.6 (22.4)	n.s.^#^
Survival from T1 [months]	22.1 (18.9)	23.1 (19.1)	21.4 (18.8)	N.S.^§^	24.3 (20.4)	15.9 (11.8)	0.015^§^	28.3 (20.8)	17.6 (11.5)	18.4 (21.9)	< 0.001^§^
Survival from T0 [months]	52.4 (40.8)	56 (48.3)	49.3 (33.3)	N.S.^§^	54.7 (42.5)	45.6 (35.2)	N.S.^§^	71.9 (50.6)	39.7 (15.4)	27.4 (21.9)	< 0.001^§^

Numbers are depicted as mean and standard deviation (in brackets), or absolute number/percentage (in relation to the subgroup specified at the top of each column)

*p* values ≤ 0.05 were considered significant. *n.s.* not significant; ^§^, Mann–Whitney *U* test or Kruskal–Wallis test as appropriate; ^#^, *t* test or ANOVA as appropriate

Post-hoc analyses:

*EMBE:* slow vs. moderate progressors, *p* = 0.004; slow vs. fast progressors, *p* = 0.003; moderate vs. fast progressors, n.s.

*Survival from T1:* slow vs. moderate progressors, *p* = 0.003; slow vs. fast progressors, *p* = 0.001; moderate vs. fast progressors, n.s.

*Survival from T0*: slow vs. moderate progressors, *p* < 0.001; slow vs. fast progressors, *p* < 0.001; moderate vs. fast progressors, *p* < 0.001

Progression rate was available in 151 patients. T0, date of symptom onset; T1, date of death or date of the last clinical status report for patients still alive

### Ventilatory support

Between T1 and T2, NIV was eventually initiated in 108/158 (68.4%) patients. In 77 subjects, NIV was started shortly after T1, and 31 patients went on NIV later during the disease course. Following baseline sleep studies, initiation of NIV was based on the diagnosis of either SDB (*n* = 63) or on FVC reduction and symptoms of respiratory muscle weakness only (*n* = 14). Regular use of NIV until T2 was documented for 95/158 patients, referred to as the NIV(+) group. The NIV(–) group (63/158 patients) comprised 47 subjects in whom ventilatory support was never established, 13 patients who aborted NIV without later recommencing it, and 3 patients with isolated OSA on initial sleep studies who used nocturnal CPAP therapy until T2. In patients who were deceased by the time of database closure (*n* = 105) NIV had been regularly used until death by 71 individuals (67.6%), while this was the case among 24/53 patients (45.3%) who were still alive.

### Survival from diagnostic sleep studies (T1)

Survival analyses focused on the impact of respiratory sleep outcomes in either NIV(+) or NIV(–) patients. We investigated mean survival, cumulative survival and hazard ratios with regard to specific thresholds for parameters reflecting SDB. In addition, bivariate correlation analysis and linear regression were used to test for associations between continuous data and survival.

Mean survival time after T1 was 22.1 (18.9) months among all patients, 23.8 (20.4) months in the NIV(+) group and 19.6 (16.1) months in the NIV(–) group, with no significant group difference (Table 1).

In the NIV(–) group, patients in whom maximum *p*_tc_CO_2_, AHI, or EMBE at T1 surpassed the specific cut-off value (≥ 50 mmHg, ≥ 5/h, or > 2 mmol/l) showed significant reduction of mean survival time compared to patients with sub-threshold values (Table 3). In contrast, when patients in the NIV(+) group were stratified accordingly, mean survival time did not differ between subgroups. Thus, once NIV was established and steadily used, the negative impact of baseline respiratory measures on mean life span was abolished. Notably, NIV(+) patients in whom the AHI had been ≥ 5/h on baseline sleep studies showed longer median survival than subjects with an initial AHI < 5/h although this difference missed statistical significance (25.1 ± 20.3 months vs. 22.1 ± 20.7 months).

**Table 3:** Survival from baseline sleep studies (time point T1)

Parameter	NIV status	Stratification	*n*	Mean survival (months)	Median survival (months)	*p*
AHI	NIV(+)	< 5/h	40	22.1 (20.7)	17.9 (0.5–99.2)	n.s.
		≥ 5/h	55	25.1 (20.3)	17.5 (2.4–97.3)	
	NIV(–)	< 5/h	38	22.7 (17.5)	19.5 (2.3–89.3)	0.027
		≥ 5/h	25	14.8 (12.8)	12.9 (0.7–46.3)	
max. p_tc_CO_2_	NIV(+)	< 50 mmHg	59	26.2 (23.1)	19.5 (0.5–99.2)	n.s.
		≥ 50 mmHg	36	20.0 (14.6)	16.9 (3.0–60.7)	
	NIV(–)	< 50 mmHg	59	20.4 (16.4)	15.7 (0.7–89.3)	0.025
		≥ 50 mmHg	4	7.3 (1.9)	7.3 (5.1–9.7)	
$t_{{{\text{CO}}_{{2}} }}$ ≥ 50	NIV(+)	< 30 min	72	24.1 (20.2)	19.1 (0.5–97.3)	n.s.
		≥ 30 min	17	20.4 (13.9)	16.9 (4.4–52.6)	
	NIV(–)	< 30 min	63	19.6 (16.1)	14.9 (0.7–89.3)	–
		≥ 30 min	0			
EMBE	NIV(+)	≤ 2 mmol/l	36	28.4 (23.4)	20.5 (2.4–97.3)	n.s.
		> 2 mmol/l	52	18.3 (11.5)	16.6 (0.5–48.1)	
	NIV(–)	≤ 2 mmol/l	30	26.5 (17.9)	24.5 (1.4–89.3)	< 0.001
		> 2 mmol/l	28	13.0 (11.1)	10.8 (1.8–56.4)	
upright FVC	NIV(+)	≥ 70% pred	25	28.7 (20.8)	26.2 (2.4–78.5)	n.s.
		< 70% pred	35	22.3 (21.7)	16.9 (0.5–99.2)	
	NIV(−)	≥ 70% pred	26	23.4 (20.7)	18.8 (2.3–89.3)	n.s.
		< 70% pred	11	19.5 (13.0)	16.1 (1.8–46.3)	

Patients were stratified according to baseline respiratory sleep outcomes (using specific cut-off values) and NIV status. Mean survival is depicted in column 5, with standard deviation in brackets. Median survival is shown in column 6 (with range in brackets)

*AHI* apnea hypopnea index, *p*_*tc*_*CO*_*2*_ maximum transcutaneous carbon dioxide tension, $t_{{{\text{CO}}_{{2}} }}$  *≥* *50* cumulative duration of nocturnal hypercapnia ≥ 50 mmHg, *EMBE* early morning base excess, *FVC* forced vital capacity, *NIV* non-invasive ventilation

*p* values in the last column refer to the Mann–Whitney *U* test

When the NIV(+) and NIV(–) groups were directly compared, patients with EMBE > 2 mmol/l or AHI ≥ 5/h at T1 showed longer survival if enduringly ventilated (EMBE: *p* = 0.022; AHI: *p* = 0.012). For subjects with maximum *p*_tc_CO_2_ ≥ 50 mmHg this finding could not be reproduced, most likely because only 4 of those patients had not started NIV.

**Fig. 1:**
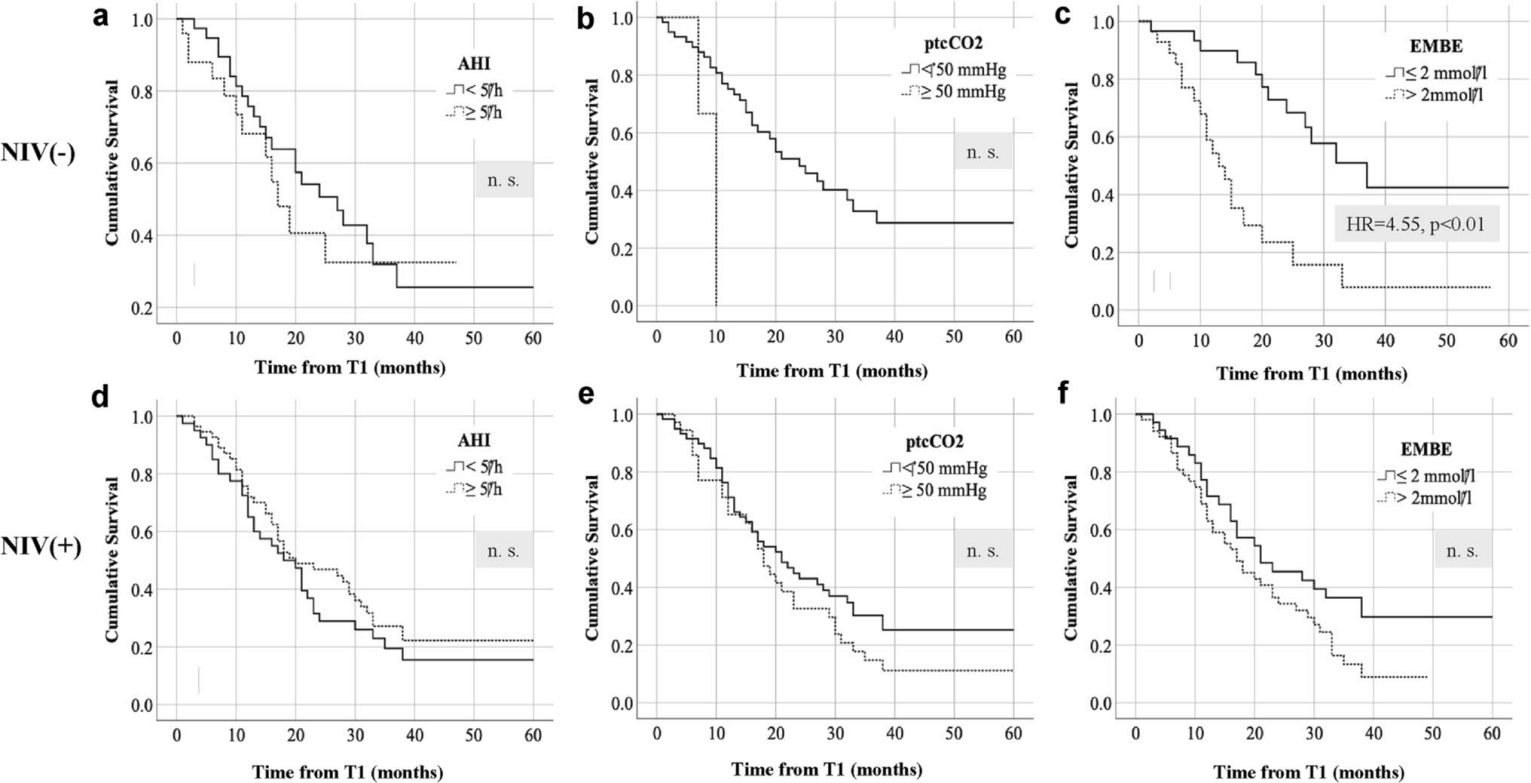
Survival from baseline sleep studies (time point T1) in patients with ALS. **a**–**c** refer to patients who did not undergo non-invasive ventilation, **d**–**f** depict Kaplan-Meyer plots for patients who started enduring NIV following T1. Survival analyses were performed using critical cut-off values for respiratory sleep outcomes at T1. *AHI* apnea hypopnea index, *EMBE* early morning base excess, *ptcCO*_*2*_ maximum nocturnal transcutaneous CO_2_ tension, *X axis* months, *Y axis* cumulative survival. *p* values < 0.05 were considered significant

Cumulative survival for different respiratory strata was visualized using Kaplan-Meyer curves (Fig. 1). In NIV(–) patients, an increased hazard ratio was only found for EMBE > 2 mmol/l (4.55, *p* < 0.01). In patients who subsequently underwent NIV no respiratory parameter was associated with an increased hazard ratio when the respective cut-off value was surpassed (Fig. 1).

Bivariate analysis in the NIV(–) group revealed only weak inverse correlation of survival from T1 with maximum *p*_tc_CO_2_ (*r* = − 0.25; p = 0.050), $t_{{{\text{CO}}_{{2}} }}$_≥ 50_ (*r* = − 0.28; *p* = 0.027), and AHI (*r* = − 0.26; *p* = 0.042) but markedly stronger negative correlation with EMBE (*r* = − 0.53; *p* < 0.001). In the NIV(+) group, both EMBE and FVC were weakly associated with survival time (EMBE: *r* = − 0.28, p = 0.008; FVC: *r* = 0.31, *p* = 0.015).

Linear regression identified EMBE as the only independent significant predictor of survival after T1 in NIV(–) and NIV(+) patients (Supplemental Table 1).

### Survival from symptom onset (T0)

**Fig. 2:**
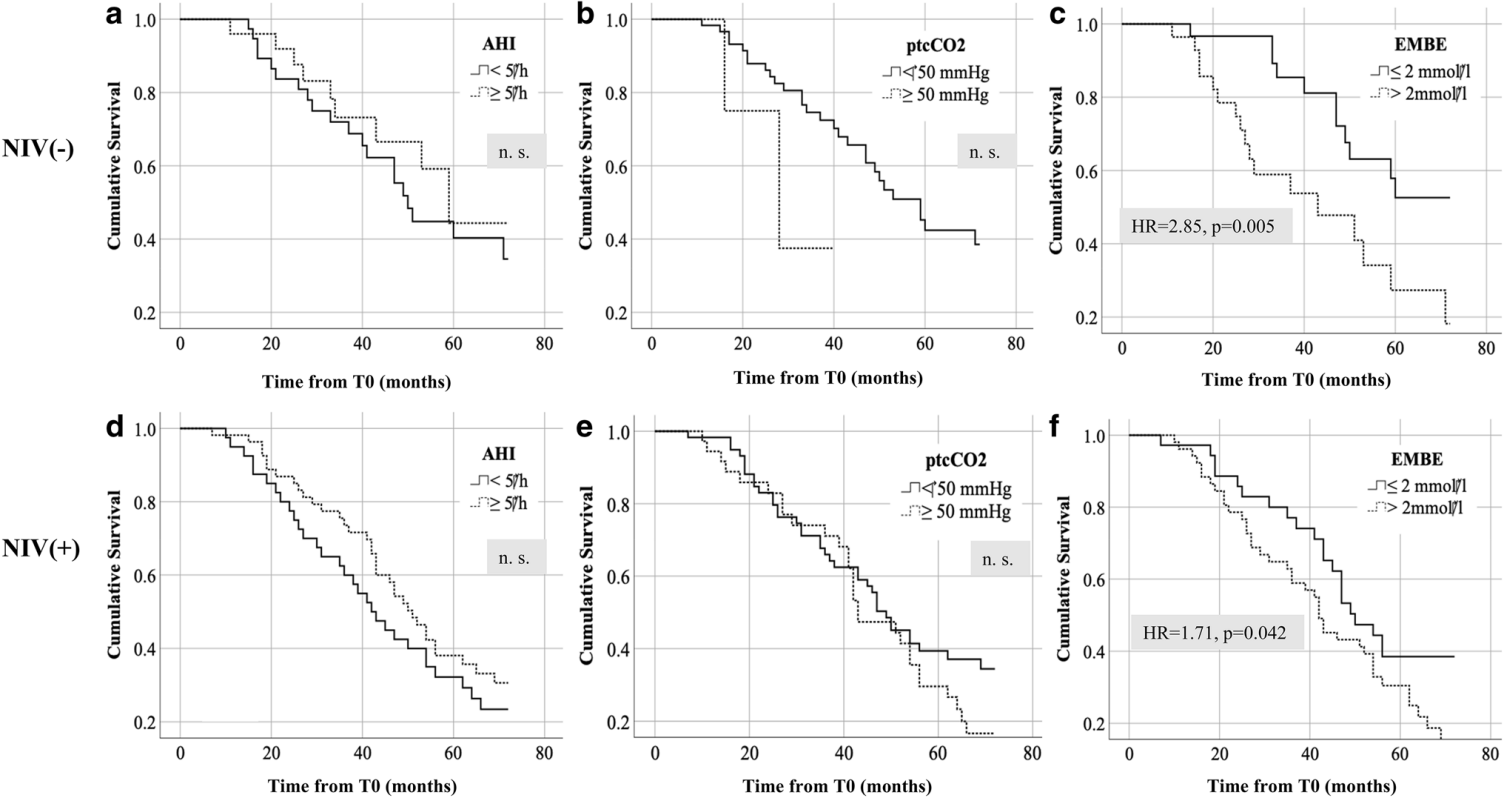
Survival from symptom onset (time point T0). Survival analyses were performed using critical cut-off values for respiratory sleep outcomes at baseline sleep studies (time point T1). **a**–**c** Refer to patients who did not undergo non-invasive ventilation (NIV), **d**–**f** depict Kaplan-Meyer plots for patients who started enduring NIV following T1. *AHI* apnea hypopnea index, *EMBE* early morning base excess, *ptcCO*_*2*_ maximum nocturnal transcutaneous CO_2_ tension, *X axis* months, *Y axis* cumulative survival. *p* values < 0.05 were considered significant

Regarding survival from T0 the same statistical approach and threshold values were used as described above. Mean survival time after T0 was 52.4 (40.8) months among all patients, 50.8 (31.1) months in the NIV(+) group and 54.8 (52.4) months in the NIV(–) group, with no significant group differences (Table 1). Mean survival times are depicted in Table 4. In NIV(–) patients, reduction of mean survival was only found in association with baseline EMBE > 2.0 mmol/l but not with any of the other respiratory measures. The same effect of EMBE > 2 mmol/l was not observed in NIV(+) patients. Cumulative survival for different respiratory strata was again visualized using Kaplan-Meyer curves (Fig. 2). Increased hazard ratios were only found for EMBE > 2 mmol/l in both ventilated and non-ventilated patients, with relative risk increase being markedly higher in the NIV(–) subgroup (NIV(–): 2.85, *p* = 0.005; NIV(+): 1.71, *p* = 0.042).

**Table 4:** Survival from symptom onset (time point T0)

Parameter	NIV status	Stratification	n	Mean survival (months)	Median survival (months)	*p*
AHI	NIV(+)	< 5/h	40	46.1 (26.2)	41.6 (9.6–128.2)	n.s.
		≥ 5/h	55	54.1 (34.1)	48.7 (6.0–173.1)	
	NIV(−)	< 5/h	38	51.0 (40.1)	42.2 (14.3–223.0)	n.s.
		≥ 5/h	25	60.5 (67.5)	36.2 (10.1–341.1)	
max. *p*_tc_CO_2_	NIV(+)	< 50 mmHg	59	54.0 (34.6)	47.0 (6.0–173.1)	n.s.
		≥ 50 mmHg	36	45.4 (23.9)	423 (9.6–104.4)	
	NIV(–)	< 50 mmHg	59	56.7 (53.5)	42.0 (10.1–341.1)	n.s.
		≥ 50 mmHg	4	25.6 (10.2)	23.6 (16.0–39.2)	
$t_{{{\text{CO}}_{{2}} }}$ ≥ 50	NIV(+)	< 30 min	72	52.0 (32.1)	47.0 (6.0–173.1)	n.s.
		≥ 30 min	17	44.9 (22.2)	42.0 (14.0–99.2)	
	NIV(−)	< 30 min	63	54.8 (52.4)	39.1 (10.1–341.1)	–
		≥ 30 min	0			
EMBE	NIV(+)	≤ 2 mmol/l	36	58.2 (36.9)	47.9 (6.0–173.1)	n.s.
		> 2 mmol/l	52	43.2 (23.7)	41.5 (9.6–128.1)	
	NIV(–)	≤ 2 mmol/l	30	67.7 (61.8)	49.5 (14.3–341.1)	0.007
		> 2 mmol/l	28	41.5 (39.5)	29.4 (10.1–223.0)	
upright FVC	NIV(+)	≥ 70% pred	25	51.0 (32.2)	46.9 (6.0–128.1)	n.s.
		< 70% pred	35	52.8 (34.5)	48.7 (9.6–173.1)	
	NIV(−)	≥ 70% pred	26	47.1 (34.5)	38.0 (10.1–144.2)	n.s.
		< 70% pred	11	48.2 (27.7)	42.0 (16.0–117.1)	

Patients were stratified according to baseline respiratory sleep outcomes (using specific cut-off values) and NIV status. Mean survival is depicted in column 5, with standard deviation in brackets. Median survival is shown in column 6 (with range in brackets)

*EMBE* early morning base excess, *FVC* forced vital capacity, *AHI* apnea hypopnea index, *p*_*tc*_*CO*_*2*_ maximum transcutaneous carbon dioxide tension, $t_{{{\text{CO}}_{{2}} }}$  *≥ 50* cumulative duration of nocturnal hypercapnia ≥ 50 mmHg

*p* values in the last column refer to the Mann–Whitney *U* test

Linear regression with AHI, EMBE, maximum *p*_tc_CO_2_, $t_{{{\text{CO}}_{{2}} }}$_≥ 50_ and ALS-FTD integrated into the model showed that only EMBE at T1 independently predicted survival after T0 in both NIV(–) and NIV(+) patients (Supplemental Table S2). The presence of ALS-FTD significantly predicted survival in NIV(–) patients only (data not shown).

## Discussion

The present study investigated whether respiratory parameters on baseline sleep studies impact survival in patients with ALS. Only few previous studies had a similar purpose, either focusing on sleep apnea or daytime blood gas analysis, but without taking into account whether enduring NIV was subsequently established [14, 18]. In one study, overall prognosis appeared to be worse in ALS patients with concomitant OSA but indication for NIV was based on the AHI alone, and adherence to NIV was not considered [14]. Recently, it was reported that ALS patients with normal *p*CO_2_ and bicarbonate values on daytime blood gas analysis show longer survival than patients with normal *p*CO_2_ and increased bicarbonate or patients with elevation of both parameters [18]. To both underline and complement this finding the following conclusions can be drawn from the present study:

1. EMBE > 2 mmol/l reliably predicts nocturnal hypercapnia. This finding is in line with a previous study showing that daytime bicarbonate levels indicate respiratory muscle weakness and SDB even if daytime *p*CO_2_ is lower than the values on which prescription of NIV is usually based on [18]. Of note, > 2 mmol/l as a cut-off value for daytime base excess is markedly lower than the thresholds previously proposed regarding patients with ALS or Duchenne’s muscular dystrophy [10, 32].
2. Nocturnal hypercapnia on baseline sleep studies predicts shorter survival. Evolving hypoventilation as reflected by maximum nocturnal *p*_tc_CO_2_ ≥ 50 mmHg or EMBE > 2 mmol/l is likely to be indicative of more advanced and aggressive disease. Accordingly, EMBE was correlated with ΔFS at T1 (*r* = 0.313; *p* < 0.000) and possibly parallels disease progression. The negative effect of EMBE > 2 mmol/l on survival was markedly higher in the NIV(–) than the NIV(+) group. As base excess is not yet part of standard criteria for NIV indication almost one third of patients with EMBE > 2 mmol/l were not started on ventilatory support. Since this subgroup showed the highest hazard ratio for shorter survival we postulate that EMBE should be considered in NIV indication for patients with ALS at least. Further studies are necessary to evaluate EMBE in other neuromuscular conditions with less rapid disease progression.
3. Reduction of FVC is closely associated with shorter survival in patients with ALS [33]. The present study shows that life span is also reduced in patients who surpass critical values for maximum *p*_tc_CO_2_, AHI and EMBE on diagnostic sleep studies and do not subsequently start NIV. In contrast, the negative impact of SDB on remaining life span is abolished by regular usage of NIV. This finding underlines that survival analyses in ALS should take ventilatory support into account. Interestingly, patients with OSA even lived longer than patients without OSA once NIV was established. The presence of OSA at baseline might indicate better diaphragm strength (still sufficient to collapse the upper airway during inspiration) which possibly explains why NIV(+) patients with OSA showed longer survival than subjects without OSA. In contrast, OSA was related to reduced survival in NIV(–) patients which may reflect a negative impact that is independent of diaphragm function.
4. This study suggests that early initiation of NIV relates to longer survival although it was not specifically designed to test this hypothesis as a previous study by Vitacca et al. [8]. Quantitative measures indicating SDB were inversely related with life span in non-ventilated patients, and this association was abrogated following NIV initiation. As respiratory failure is known to evolve in a continuum ranging from CO_2_ retention during REM sleep to daytime hypercapnia [9] it can be assumed that survival benefits from NIV depend on the point of treatment start.
5. As shown by previous studies, adequate follow-up of ventilated patients requires titration of ventilator settings to sustainably achieve normocapnia, normoxia, and normalization of the AHI [19–21]. In the present study, it was attempted to meet this goal in routine practice. Since linear regression analysis showed that EMBE > 2 mmol/l independently predicts survival after baseline sleep studies also in NIV(+) patients, it is desirable to further assess whether adjustment of NIV settings should also aim to correct EMBE below 2 mmol/l.
6. Lastly, the present study suggests that prior attempts to specifically define sleep-related hypoventilation by duration of nocturnal hypercapnia were arbitrary. Based on transcutaneous capnography, two national guidelines proposed thresholds of either ≥ 10 min (*p*_tc_CO_2_ ≥ 55 mmHg) or ≥ 30 min (*p*_tc_CO_2_ ≥ 50 mmHg) [24, 26]. For both numbers no published evidence is available, and they were presumably meant to take into account that transcutaneous capnometry is somewhat inaccurate. We specifically investigated the 30-min cut-off in patients with ALS and did not find that surpassing it was specifically related to shorter survival even in non-ventilated patients. We conclude that the 30-min threshold is not helpful for guiding treatment decisions and may even get in the way when it comes to early indication for NIV in patients with ALS.

### Study limitations

It might be considered a limitation of this study that death and tracheostomy were not combined to form a common endpoint. Furthermore, patients who underwent tracheostomy were not even assigned to the NIV(+) group. This was avoided for several reasons: The number of tracheostomized patients was too small (16/158) for valid statistical analysis, and in most cases, it was impossible to retrospectively identify whether tracheostomy was performed due to respiratory or bulbar deterioration. In addition, tracheostomy may substantially prolong survival to a point that patients would probably not have reached with ongoing NIV [34]. Thus, inclusion of patients with invasive ventilation would possibly have confounded survival time in the NIV(+) group. Lastly, patients may gain meaningful prolongation of life span and also quality of life from invasive ventilation, rendering it inappropriate to generally equate tracheostomy with death.

It has to be acknowledged that in a subset of patients, overall survival and the use of NIV may be negatively affected by cognitive and behavioral impairment and overt ALS-FTD, in particular. However, the present study was not designed to specifically investigate this aspect, and the number of patients fulfilling diagnostic criteria for ALS-FTD was rather small.

Further limitations comprise the retrospective study design and the fact that some clinical information was only available from deceased patients’ dependents. Moreover, the number of patients with sleep-related hypoventilation who did not start NIV was extremely small, hampering further statistical analysis regarding this subgroup. Notably, all patients with $t_{{{\text{CO}}_{{2}} }}$_≥ 50_ > 30 min subsequently underwent NIV.

To conclude, the present study evaluated the impact of SDB and NIV on survival in patients with ALS. It underlines the importance of transcutaneous capnography for diagnostic and prognostic purposes, and strongly suggests that serum bicarbonate (or EMBE, respectively) does not only predict respiratory muscle weakness and SDB as previously shown [18] but also survival. Most importantly, this study suggests that the specific impact of SDB on overall prognosis can be neutralized by implementation of enduring NIV. Once indicated NIV leads to a meaningful prolongation of life span. In this sense, the findings presented here add to an increasing body of evidence showing that for patients with ALS, NIV is actual treatment rather than mere palliation.

## Supplementary Information

Below is the link to the electronic supplementary material.

Supplementary file1 (DOCX 15 KB)

Supplementary file2 (DOCX 14 KB)

## Abbreviations

AHI: Apnea hypopnea index
ALS: Amyotrophic lateral sclerosis
ALS-FRS-R: Revised ALS Functional Rating Scale
CPAP: Continuous positive airway pressure
EMBE: Early morning base excess
EPAP: Expiratory positive airway pressure
FVC: Forced vital capacity
NIV: Non-invasive ventilation
ODI: Oxygen desaturation index
OSA: Obstructive sleep apnea
p_tc_CO_2_: Transcutaneous carbon dioxide tension
SDB: Sleep-disordered breathing
SpO_2_: Peripheral oxygen saturation
T0: Date of self-reported symptom onset
T1: Date of baseline sleep evaluation
T2: Date of death for deceased patients or date of last clinical status report
$t_{{{\text{CO}}_{2} }}$_≥__50_: Cumulative duration of p_tc_CO_2_ ≥ 50 mmHg
$t_{{{\text{SpO}}_{2} }}$_<__90_: Duration of SpO_2_ falling below 90%
ΔFS: Linear progression rate; 48 − ALS-FRS-R sum score/duration from disease onset in months
