# Supplementary material for: Respiratory parameters on diagnostic sleep studies predict survival in patients with amyotrophic lateral sclerosis
Source: J Neurol. 2021 Apr 20;268(11):4321–31. doi: 10.1007/s00415-021-10563-0 (PMC8505303; doi:10.1007/s00415-021-10563-0)
Supplement: Supplementary file 1 — Supplementary file1 (DOCX 15 KB) [file 415_2021_10563_MOESM1_ESM.docx]

|  | NIV(+) (n=82) | NIV(-) (n=57) |
| --- | --- | --- |
| R^2^ | 0.138 | 0.254 |
| ANOVA | p=0.020 | p=0.004 |
| AHI | b=0.071 (n. s.) | b=-0.213 (n. s.) |
| max. p_tc_CO_2_ | b=0.483 (n. s.) | b=0.113 (n. s.) |
| t_CO2≥50_ | b=-0.149 (n. s.) | b=-0.173 (n. s.) |
| EMBE | b=-0.546 (p=0.001) | b=-0.484 (p=0.001) |

Supplemental Table S1: Summarized linear regression models for survival after baseline sleep studies (time point T1). ANOVA, analysis of variance, AHI, apnea hypopnea index, NIV, non-invasive ventilation, NIV(+), patients with sustained usage of non-invasive ventilation, ptcCO2, transcutaneous carbon dioxide tension, t_CO2≥50,_, cumulative duration of ptcCO2 ≥50 mmHg, NIV(-), patient who did not undergo NIV, b, standardized regression coefficient; p values ≤ 0.05 were considered significant; n. s., not significant.

To fulfill criteria for linear regression, 2 patients with survival after T1 ≥ 80 months were not integrated into the model. In 17 individuals EMBE or t_CO2≥50_ were not available.
