# Supplementary material for: Respiratory parameters on diagnostic sleep studies predict survival in patients with amyotrophic lateral sclerosis
Source: J Neurol. 2021 Apr 20;268(11):4321–31. doi: 10.1007/s00415-021-10563-0 (PMC8505303; doi:10.1007/s00415-021-10563-0)
Supplement: Supplementary file 2 — Supplementary file2 (DOCX 14 KB) [file 415_2021_10563_MOESM2_ESM.docx]

|  | NIV(+) (n=83) | NIV(-) (n=56) |
| --- | --- | --- |
| R^2^ | 0.169 | 0.146 |
| ANOVA | p=0.006 | N.S. (p=0.084) |
| AHI | b=0.139 (n. s.) | b=-0.108 (n. s.) |
| max. p_tC_CO_2_ | b=0.120 (n. s.) | b=0.133 (n. s.) |
| t_CO2≥50_ | b=0.089 (n. s.) | b=-0.178 (n. s.) |
| EMBE | b=-0.518 (p= 0.001) | b=-0.386 (p= 0.011) |

Supplemental Table S1: Summarized linear regression models for survival after symptom onset (time point T0). ANOVA, analysis of variance, AHI, apnea hypopnea index, NIV, non-invasive ventilation, NIV(+), patients with sustained usage of non-invasive ventilation, ptcCO2, tanscutaneous carbon dioxide tension, t_CO2≥50,_, cumulative duration of ptcCO2 ≥50 mmHg, NIV(-), patient who did not undergo NIV, b, standardized regression coefficient; p values ≤ 0.05 were considered significant; n. s., not significant.

To fulfill criteria for linear regression, 2 patients with survival after T0 ≥ 220 months were not integrated into the regression model. In 17 individuals EMBE or t_CO2≥50_ were not available.
